# Movement Protein Pns6 of *Rice dwarf phytoreovirus* Has Both ATPase and RNA Binding Activities

**DOI:** 10.1371/journal.pone.0024986

**Published:** 2011-09-20

**Authors:** Xu Ji, Dan Qian, Chunhong Wei, Gongyin Ye, Zhongkai Zhang, Zujian Wu, Lianhui Xie, Yi Li

**Affiliations:** 1 State Key Laboratory of Protein and Plant Gene Research, Peking-Yale Joint Center for Plant Molecular Genetics and Agrobiotechnology, College of Life Sciences, Peking University, Beijing, People's Republic of China; 2 State Key Laboratory of Rice Biology, Institute of Insect Sciences, Zhejiang University, Hangzhou, People's Republic of China; 3 Biotechnology and Genetic Resources Institute, Yunnan Academy of Agricultural Sciences, Kunming, People's Republic of China; 4 Key Laboratory of Plant Virology of Fujian Province, Institute of Plant Virology, Fujian Agriculture and Forestry University, Fuzhou, People's Republic of China; University of Kansas Medical Center, United States of America

## Abstract

Cell-to-cell movement is essential for plant viruses to systemically infect host plants. Plant viruses encode movement proteins (MP) to facilitate such movement. Unlike the well-characterized MPs of DNA viruses and single-stranded RNA (ssRNA) viruses, knowledge of the functional mechanisms of MPs encoded by double-stranded RNA (dsRNA) viruses is very limited. In particular, many studied MPs of DNA and ssRNA viruses bind non-specifically ssRNAs, leading to models in which ribonucleoprotein complexes (RNPs) move from cell to cell. Thus, it will be of special interest to determine whether MPs of dsRNA viruses interact with genomic dsRNAs or their derivative sRNAs. To this end, we studied the biochemical functions of MP Pns6 of *Rice dwarf phytoreovirus* (RDV), a member of *Phytoreovirus* that contains a 12-segmented dsRNA genome. We report here that Pns6 binds both dsRNAs and ssRNAs. Intriguingly, Pns6 exhibits non-sequence specificity for dsRNA but shows preference for ssRNA sequences derived from the conserved genomic 5′- and 3′- terminal consensus sequences of RDV. Furthermore, Pns6 exhibits magnesium-dependent ATPase activities. Mutagenesis identified the RNA binding and ATPase activity sites of Pns6 at the N- and C-termini, respectively. Our results uncovered the novel property of a viral MP in differentially recognizing dsRNA and ssRNA and establish a biochemical basis to enable further studies on the mechanisms of dsRNA viral MP functions.

## Introduction

Cell-to-cell movement is required for both local and systemic infection by plant viruses. Viruses encode movement proteins (MP) to facilitate such movement. The specific movement mechanisms vary among viruses. Some viruses, such as *Tobacco mosaic virus*, encode MPs that can alter the size exclusion limit (SEL) of plasmodesmata [1] and bind RNAs [2], and may thus move as ribonucleoprotein complexes [3]. Other viruses, including *Grapevine fanleaf virus*, move through tubular structures formed inside modified plasmodesmata that are induced by the viral MPs [4]. Many MPs are multifunctional. The 25 K MP (TGBp1, a triple gene block component) encoded by *Potato virus X* (PVX), has RNA binding, RNA helicase and Mg^2+^-dependent ATPase activities in vitro [5], [6]. It can increase the SEL of plasmodesmata in trichome cells of *Nicotiana clevelandii* [7]. It also functions as an RNA silencing suppressor that is important for PVX spreading within a host plant [8], [9]. A recent study indicated that the 25 K protein interacts with Argonaute proteins (AGO 1–4) and mediates their degradation through the proteasome pathway [10].

*Rice dwarf phytoreovirus* (RDV) is a member of the genus *Phytoreovirus* within the family Reoviridae that replicates in both host plants and insect vectors. The genome of RDV is composed of 12 double-stranded RNAs (dsRNAs) [11]. The sense strand RNAs from all genome segments of RDV contain a 5′ terminal consensus sequence (5′ GGCAAA--- or 5′ GGUAAA---) and a 3′ terminal consensus sequence (---UGAU 3′ or ---CGAU 3′) [12]. Many other reoviruses have similar sequences at the ends of their genomic dsRNAs [13], [14]. Although such sequence conservation suggests its functional significance, the biological role of these 5′- and 3′-terminal conserved sequences for RDV and other reoviruses remain unclear. In rotavirus, another member of the family Reoviridae, the terminal sequences of mRNAs form the minimal cis-acting signal required for minus-strand synthesis [15], [16].

The RDV virion has an outer shell composed of structural proteins P2, P8 and P9, and a core composed of structural proteins P1, P3, P5 and P7 as well as the genomic dsRNAs. The functions of nonstructural proteins Pns4, Pns6, Pns10, Pns11 and Pns12 have previously been characterized. Pns4 can be phosphorylated *in vivo* and is localized at the periphery of viroplasms [17]. Pns6 is involved in cell-to-cell movement [18]. Pns10 is an RNA silencing suppressor in plants [19], [20], [21] and is involved in the formation of tubular structures between neighboring insect cells [22]. Pns11 is a nucleic acid-binding protein [23]. Pns12 is essential for the formation of cytoplasmic inclusions and is involved in virion assembly [24]. Utilizing a complementation approach, Li et al. (2004) determined that Pns6, encoded by segment S6, is responsible for the movement of RDV between cells and can restore the cell-to-cell movement ability of a PVX 25 K deletion mutant [18]. In addition, Pns6 was shown to localize to plasmodesmata in epidermal cells of both *Nicotiana tabacum* bombarded and RDV-infected rice leaves. However, Pns6 did not suppress RNA silencing in cells [19].

To advance our understanding of the cell-to-cell movement mechanisms of dsRNA viruses, we studied the biochemical functions of RDV Pns6. We report here that Pns6 binds both dsRNAs and ssRNAs. Intriguingly, Pns6 exhibits non-sequence specificity for dsRNA but shows preference for ssRNA sequences derived from the conserved genomic 5′- and 3′- terminal consensus sequences of RDV. Furthermore, Pns6 exhibits magnesium-dependent ATPase activities. Mutagenesis identified the Pns6 RNA binding and ATPase activity sites at the N- and C-termini, respectively. Our results uncovered the novel property of a viral MP in differentially recognizing dsRNA and ssRNA and establish a biochemical basis to enable further studies on the mechanisms of dsRNA viral MP functions.

## Results

### Sequence analysis of Pns6 reveals potential RNA acid binding region and ATPase/helicase motif

**Figure 1:**
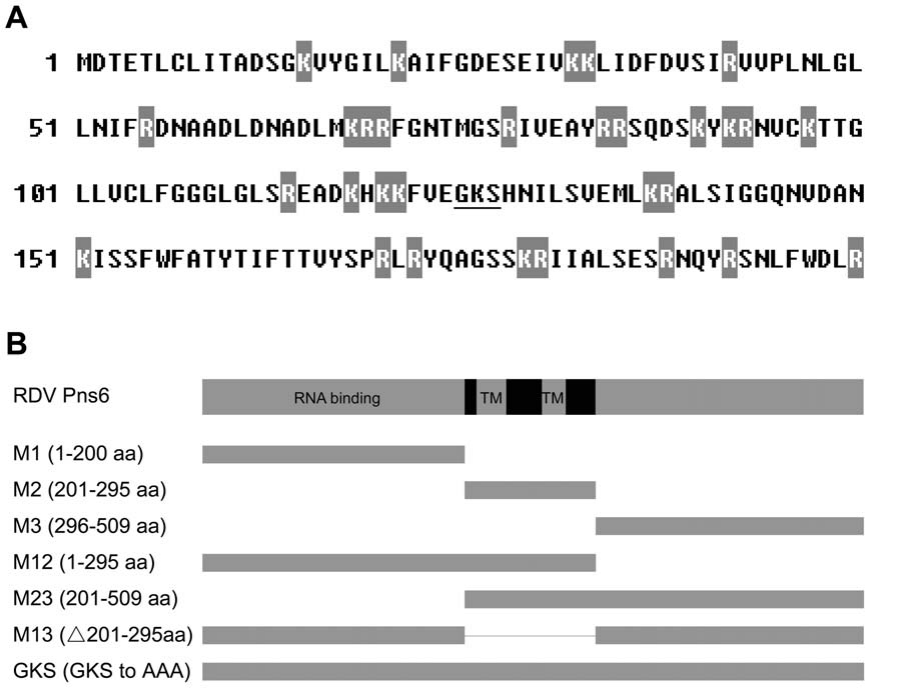
Analysis of Pns6 sequence and construction of mutants. (A) The N terminal region of Pns6 is rich in basic amino acids. Lysines (K) and arginines (R) are indicated in white font on a gray background, and the GKS motif is underlined. (B) The N-terminus of Pns6 is predicted to contain the RNA binding region and its middle region is predicted to contain transmembrane domains (TM). M1-M13 indicate deletion mutants with amino acid sequences shown in parentheses. In the GKS mutant, the amino acids GKS at positions125–127 are replaced with AAA.

In our previous study, Pns6 was shown to restore the cell-to-cell movement activity of a PVX 25 K deletion mutant [18]. The 25 K protein of PVX is a multifunctional protein that contains different functional domains [25]. We predicted the potential functional domains of Pns6 based on amino acid sequence. As Figure 1A shows, the N-terminal region of Pns6 is rich in basic amino acids, potentially containing an RNA-binding site. In the internal region of Pns6, two transmembrane helicase domains were predicted at amino acid positions 207–228 and 254–271. A putative GKS motif, potentially related to ATPase/helicase activity, was present at amino acid positions 125–127 (Figure 1B).

### Pns6 binds both single-stranded and double-stranded RNA

**Figure 2:**
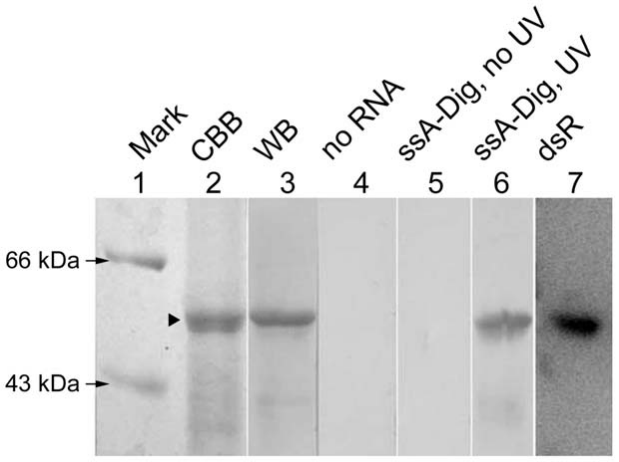
Northwestern blotting showing Pns6 binding to ssRNA and dsRNA. Lanes 1 and 2: Coomassie Brilliant Blue staining. “Mark” indicates the protein molecular standards, the arrowheads indicate 66 kDa and 43 kDa bands. The black triangle indicates the His-Pns6 band. Lane 3: His-Pns6 was detected by western blotting (WB) with anti-His antibody. Lane 6: after incubation and UV crosslinking, the digoxin-labeled single-stranded RNA probe ssA-Dig bound to His-Pns6 on the membrane was detected with anti-digoxin (AP-conjugated) antibody. Lanes 4 & 5: negative controls. Lane 7: the radiolabeled dsRNA probe dsR bound to His-Pns6 was detected after 8 hours of exposure.

**Figure 3:**
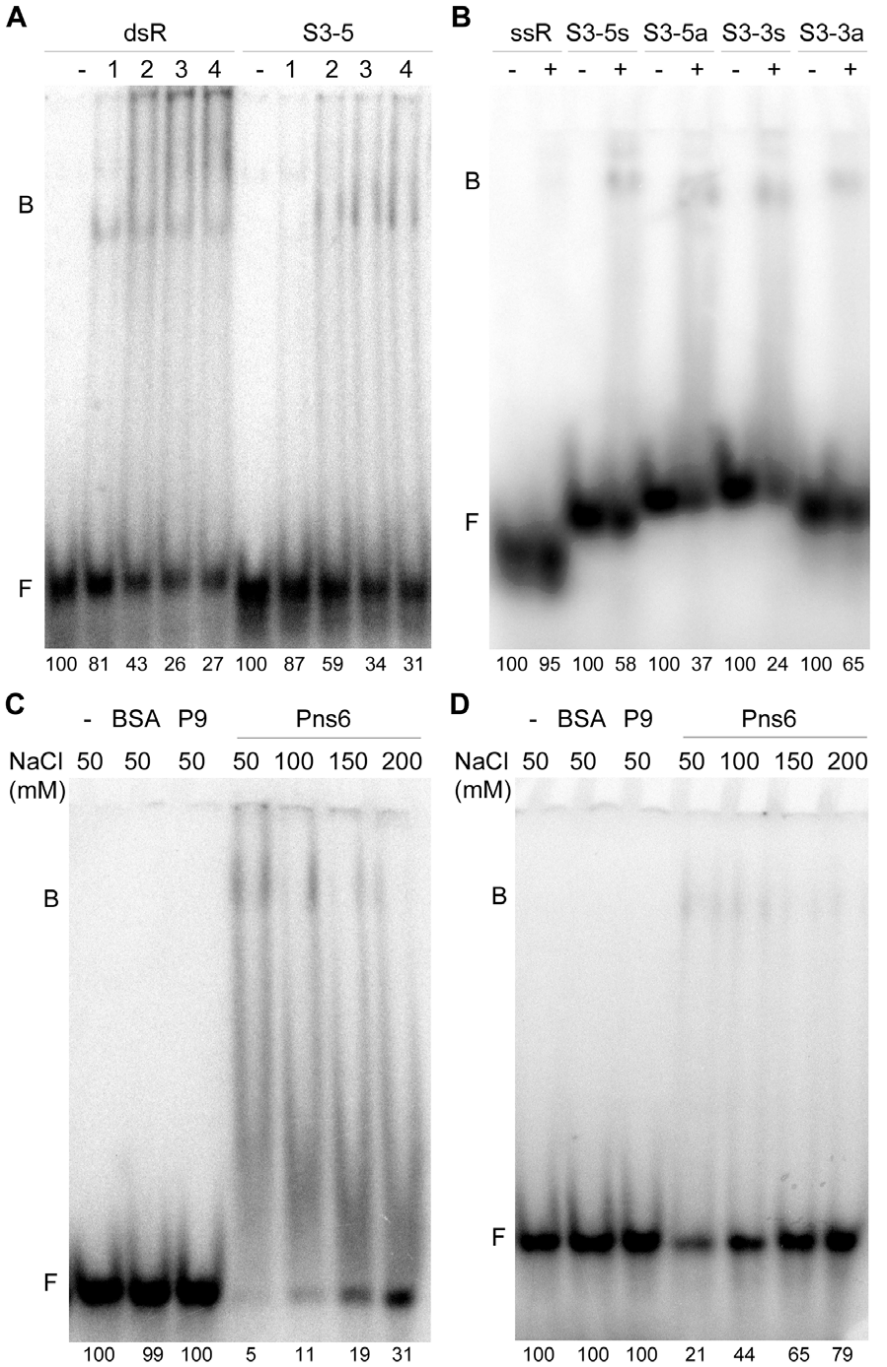
EMSA confirmation of the RNA binding ability of Pns6 and the influence of salt concentrations on the binding. (A) and (B) EMSA (electrophoresis mobility shift assays) showing Pns6 binding to dsRNA and ssRNA, respectively. And the RNA binding activities of Pns6 was affected by salt concentration. Free and bound RNA probes are indicated by ‘F’ and ‘B’, respectively. Free probes were quantified with ImageJ software version 1.4. The relative values of free probe are presented below each of the lanes. In (A) and (B) 30 nanogram (ng) of BSA was added in lanes labeled ‘−’ as a negative control. In (A), the random dsRNA (dsR) and conserved dsRNA (S3-5) were used at 5 ng, and lanes 1, 2, 3, and 4 represent His-Pns6 used at 10, 20, 30 or 40 ng, respectively. In (B), Pns6 was added at 30 ng in lanes labeled ‘+’. The random ssRNA (ssR) and conserved RDV ssRNA (S3-5s, S3-5a, S3-3s and S3-3a) were used at 2.5 ng. (C) and (D) Effects of salt concentrations on Pns6-RNA interaction. In (C) and (D), no protein was added in lanes labeled ‘−’. BSA and His-P9 served as negative controls. Proteins were added at 50 ng in each lane. In (C), the dsRNA (S3-5) was used at 2.5 ng. In (D), the ssRNA (S3-5a) was used at 2.5 ng.

During RDV replication, both dsRNAs and ssRNAs are produced. Therefore, both forms of RNA (Table 1) were used to test the RNA binding activity of Pns6. We first tested RNA probes of non-RDV sequences, including a human α-actin sequence (ssA) and a random dsRNA sequence (dsR), in northwestern blotting assays. Figure 2 shows that Pns6 could bind both probes. We then performed electrophoresis mobility shift assay (EMSA) to further confirm the RNA binding activity of Pns6. As shown in Figure 3A, Pns6 bound dsR as well as an RDV-specific sequence (S3-5) with equal efficiency. Pns6 also bound ssRNAs (Figure 3B). However, binding to a random ssRNA sequence (ssR) was weaker than binding to RDV-specific ssRNA sequences (S3-5s, S3-5a, S3-3s and S3-3a). These data establish that Pns6 has RNA binding activities and suggest that it appears to have sequence preference for ssRNAs. This was further tested as described below.

**Table 1:** Sequences of dsRNA and ssRNA probes.

	Sequence(5′-3′)	Description
**dsRNA**		
S3-5	Sense: GGCAAAAUCG AGCGAACAAUAnti-sense: AUUGUUCGCU CGAUUUUGCC	Conserved S3 5′-end 20 bp sequence
S3-3	Sense: UGUUUUGCUU GGUUCCUGAUAnti-sense: AUCAGGAACC AAGCAAAACA	Conserved S3 3′-end 20 bp sequence
S3m	Sense: CAAUCAAUAU GCUCGCCCCAAnti-sense: UGGGGCGAGC AUAUUGAUUG	Non-conserved S3 sequence of 1541st-1560th bp of S3 ORF
dsR	Sense: UUCUCCGAACGUGUCACGUUUAnti-sense: ACGUGACACGUUCGGAGAAUU	Random 21 bp dsRNA sequence
**ssRNA**		
S3-5s	GGCAAAAUCG AGCGAACAAU	Conserved S3 5′-end 20 nt sense sequence
S3-5a	AUUGUUCGCU CGAUUUUGCC	Conserved S3 5′-end 20 nt antisense sequence
S3-3s	UGUUUUGCUU GGUUCCUGAU	Conserved S3 3′-end 20 nt sense sequence
S3-3a	AUCAGGAACC AAGCAAAACA	Conserved S3 3′-end 20 nt antisense sequence
S3mss		Denatured from S3m, 20 nt non-conserved S3 sequence
ssR	UUGUACUACACAAAAGUACUG	Random 21 nt ssRNA sequence
ssA	In vitro transcribed from pTripleScript-actin ™ (linearized with *Sal I*)	∼270 nt, *Homo sapiens* α-actin sequence
ssA-Dig	In vitro transcribed from pTripleScript-actin ™ (linearized with *Sal I*)	ssA sequence with digoxin label
ssB	In vitro transcribed from vector pSPT18 (linearized with *Hind III*)	∼50 nt, sequence of EcoR I, Sac I, BamH I, Xba I, Sal I, and Pst I
M2s	In vitro transcribed from pBS M2-T7 insertion (linearized with *Hind III*)	∼290 nt, the sense strand of mutant M2 of Pns6
M2a	In vitro transcribed from pBS M2-T3 insertion (linearized with *Hind III*)	∼290 nt, the antisense strand of mutant M2 of Pns6

Many MP-RNA complexes were disrupted at high salt concentrations [26], [27]. As shown in Figure 3C&D, Pns6 bound both dsRNA (S3-5) and ssRNA (S3-5a) in the range of 50–200 mM NaCl. The Pns6-RNA complexes underwent gradual dissociation with increasing salt concentrations, as expected of the influence of salt concentrations on protein-RNA interactions. In one control, BSA did not bind these RNAs. In another control, the RDV P9 protein, prepared in the same manner as Pns6 from recombinant *E. coli*, showed no interaction with the RNAs. These controls ruled out the possibility that the observed Pns6-RNA interaction was due to (i) non-specific electrostatic interactions or (ii) contaminating proteins from *E. coli* that bound the RNAs. Rather, they indicate that Pns6 interacted with the RNAs via specific molecular recognition. This specificity is further supported by the following experimental results showing selectivity of Pns6 for some RNA sequences and identifying the Pns6 domain for RNA binding.

### Pns6 binds preferentially ssRNAs derived from the terminal consensus sequences of RDV genome

**Figure 4:**
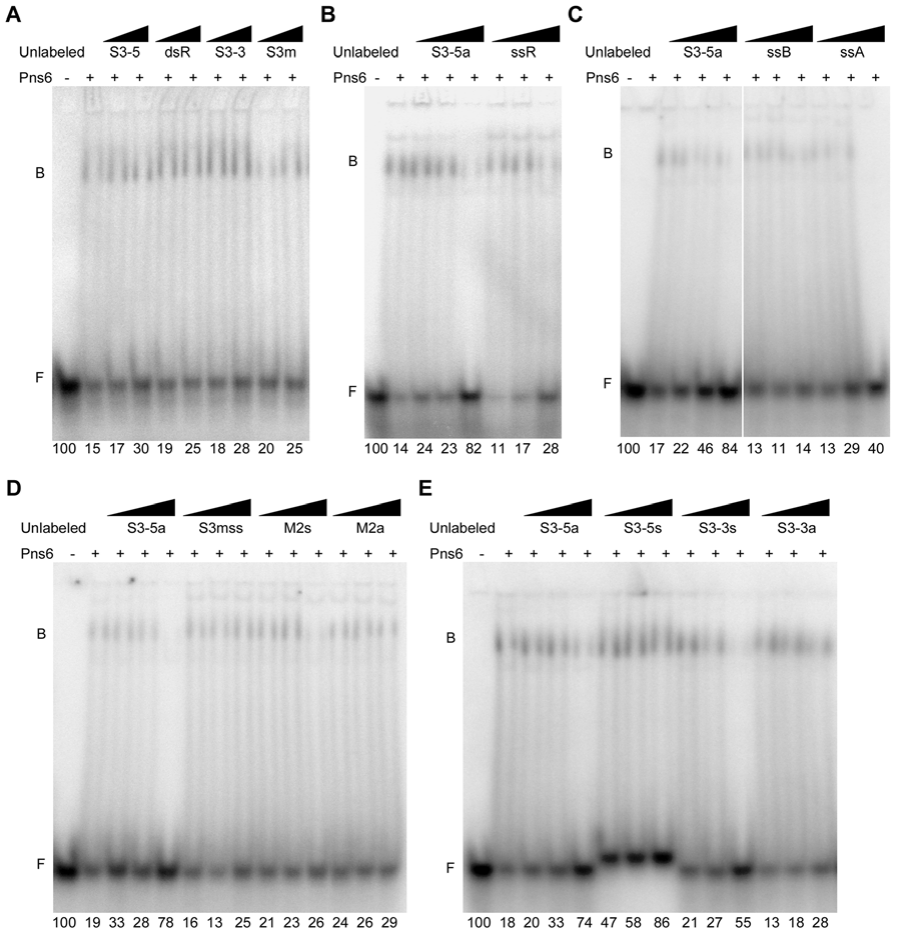
RNA binding preference for Pns6. EMSA shows Pns6 binding to dsRNA in a sequence-non-specific manner (A) and binding to ssRNA with preference for the terminal consensus sequence of the RDV genome (B–E). The labeled, conserved probes were incubated with various unlabeled competitors. The binding abilities of the competitive RNAs were measured as relative levels of free labeled probes, which were quantified with ImageJ software version 1.4. Relative values are presented below each lane. Lane ‘−’: no Pns6 and 30 ng BSA was added and all labeled probes were unbound. Lane ‘+’: 30 ng Pns6 was added. (A) Labeled, conserved dsRNA (S3-5) was added at 5 ng in each lane. Triangles indicate increasing amounts of unlabeled competitor (half or one-fold of S3-5). The four competitors had approximately equal competition abilities. (B–E) Each lane contains 2.5 ng of labeled conserved ssRNA (S3-5a). Triangles indicate increasing amounts of unlabeled competitors (one-, two- or 20-fold of S3-5a). (B) The random ssRNA (ssR) shows weaker binding to Pns6 than the S3-5a. (C) The longer random ssRNAs (ssB, about 50 nt; ssA, about 270 nt) also shows weaker binding to Pns6. (D) Three ssRNA competitors with RDV nonconserved sequences of different lengths (S3mss, M2s and M2a) show weaker binding to Pns6. (E) Among the four conserved ssRNAs (S3-5a, S3-5s, S3-3a and S3-3s), the 5′-end sense strand RDV RNAs (S3-5s) show the highest binding abilities.

Genomic RNAs of RDV consist of 12 dsRNA segments containing consensus sequences at their 5′ and 3′ ends. As described above, Pns6 showed stronger binding to ssRNA probes containing these conserved sequences than to a random ssRNA (Figure 3B). We conducted additional experiments to determine whether Pns6 has higher affinity for the terminal consensus sequences in double-stranded as well as single-stranded forms. Four unlabeled dsRNAs were used to compete with the radiolabeled conserved dsRNA S3-5 derived from RDV, including a dsRNA of random sequence (dsR), a nonconserved RDV dsRNA (S3m) and conserved dsRNAs from the 5′ and 3′ termini of RDV S3 segments (S3-5 and S3-3), respectively. The relative amounts of free labeled dsRNAs were used to determine the binding abilities of the competitors. Figure 4A shows that the four competitors had equivalent competitive abilities. The data further establish that Pns6 binds dsRNA in a sequence-non-specific manner.

To test sequence preference of ssRNA for Pns6 binding, three ssRNA competitors with random sequences and different lengths (ssR, ssB and ssA) were used to compete with a conserved ssRNA sequence (S3-5a). Figures 4B and 4C show that short random ssRNAs (21-nt ssR and 50-nt nucleotide ssB) had extremely weak competing abilities. The binding ability of the longer random ssRNA (270-nt ssA) was between that of the short random ssRNAs and that of the conserved ssRNA. Three nonconserved RDV ssRNA sequences of different lengths (S3mss, M2s and M2a) were then used to compete with S3-5a. Figure 4D shows that the binding efficiency of these competitors was similar to that of the random ssRNAs. Four ssRNAs of the RDV conserved sequences (S3-5a, S3-5s, S3-3a and S3-3s) were finally used as competitors, and they had high binding abilities (Figure 4E). S3-5s and S3-3s had higher competition efficiency than S3-5a and S3-3a (Figure 4E), suggesting that Pns6 has stronger affinity for sense-stranded RDV ssRNAs than for anti-sense ssRNAs. Furthermore, the higher binding affinity of Pns6 with S3-5a and S3-5s suggest that Pns6 had a preference for the 5′-end sense-stranded RDV RNAs. All together, these data indicate that Pns6 has preference for ssRNA derived from the terminal consensus sequences of the RDV genome.

### The RNA binding site of Pns6 is located at the N-terminal region

**Figure 5:**
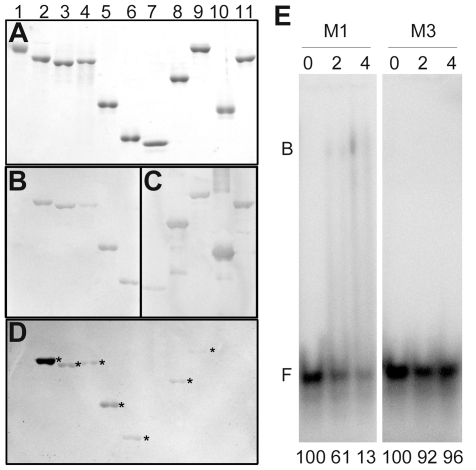
N-terminal region of Pns6 is responsible for RNA binding. (A)–(D) Northwestern blotting of mutant proteins showed that mutant M3 and all mutants containing M1 had the ability to bind ssRNA. Lane 1: BSA (negative control); 2: His-P7 (positive control); 3: His-Pns6; 4: His-GKS; 5: His-M12; 6: His-M3; 7: GST; 8: GST::M1; 9: GST::M13; 10: GST::M2; 11: GST::M23. (A) Coomassie Brilliant Blue staining; (B) western blot with anti-His antibody; (C) western blot with anti-GST antibody; (D) Northwestern blot detecting digoxin-labeled probe ssA-Dig. (E) EMSA showing dsRNA-binding activity of HisM1 and lack of such activity by HisM3. Labeled S3-5 probe was used at 5 ng in each lane. Lanes 0, 2 and 4 represent the proteins used at 0, 20 and 40 ng. Relative values of free labeled probes are presented below each lane.

The richness of basic amino acids in the N-terminal region of Pns6 suggests a role of this region in RNA binding. To test this, we generated a series of Pns6 mutants to test for RNA binding. As shown in Figure 1B, constructs M1, M2, M3, M12, M23 and M13 are deletion mutants, and the GKS construct contains a mutation at amino acids 125–127 (GKS to AAA). As shown in Figure 5A, northwestern blotting assays showed that all mutants containing the M1 region (i.e., M1, M12 and M13) as well as M3 bound ssRNA, M2 and M23 failed to bind ssRNA, and substitution of GKS with AAA had minimal effect on ssRNA binding. Furthermore, while M1 bound dsRNA of conserved RDV sequence, M3 did not exhibit clear binding activity (Fig. 5B). These results indicate that the N-terminal region of Pns6 is responsible for binding ssRNAs as well as dsRNAs.

### Pns6 is a magnesium-dependent ATPase

**Figure 6:**
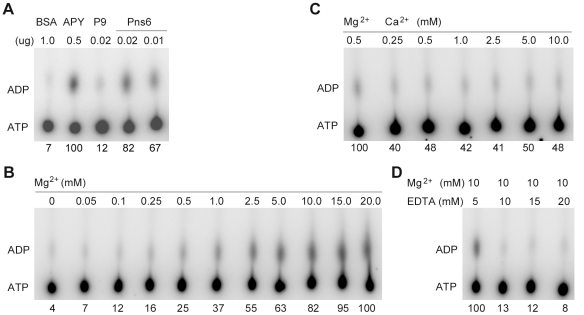
Thin layer chromatography showing Pns6 as a magnesium-dependent ATPase. (A) Hydrolysis of ATP by His-Pns6. Apyrase (APY) was used as a positive control. BSA and purified His-P9 were used as negative controls. (B) The ATPase activity of Pns6 increased with increasing concentrations of magnesium. (C) The ATPase activity of Pns6 is not dependent on calcium. (D) The ATPase activity of Pns6 was inhibited when EDTA was added at a concentration equal to or greater than that of magnesium (10 mM). In (B)–(D) His-Pns6 was used at 20 ng in each reaction. Relative values of released ADP are presented below each lane.

The putative GKS motif at amino acid positions 125 to 127 suggests that Pns6 may be an ATPase. We performed a thin layer chromatography (TLC) assay with purified His-tagged Pns6. Figure 6A shows that His-Pns6 can hydrolyze ATP into ADP and that the released ADP increased with increasing amounts of His-Pns6. This indicated that Pns6 has ATPase activity. BSA and P9 exhibited no ATPase activity, ruling out the possibility that the observed Pns6 ATPase activity was due to contaminating proteins from *E. coli*. It was previously reported that divalent cations play an important role in ATP hydrolysis reactions [28], [29]. To examine whether these ions play a role in ATP hydrolysis by Pns6, magnesium or calcium was added to the TLC assays. As shown in Figure 6B, the ATPase activity of Pns6 increased with increasing concentrations of magnesium, reaching the highest level at 5 mM of magnesium. Increasing concentrations of calcium had little effects on the ATPase activity of Pns6 (Figure 6C), indicating calcium-independence of this activity. Furthermore, when EDTA was added to the reaction system, the ATPase activity of Pns6 was efficiently inhibited, particularly when the concentration of EDTA was equal to or higher than that of magnesium (Figure 6D). These data indicate that Pns6 has ATPase activities that depend on magnesium but not calcium.

### Conserved motifs and active site of Pns6 ATPase

**Figure 7:**
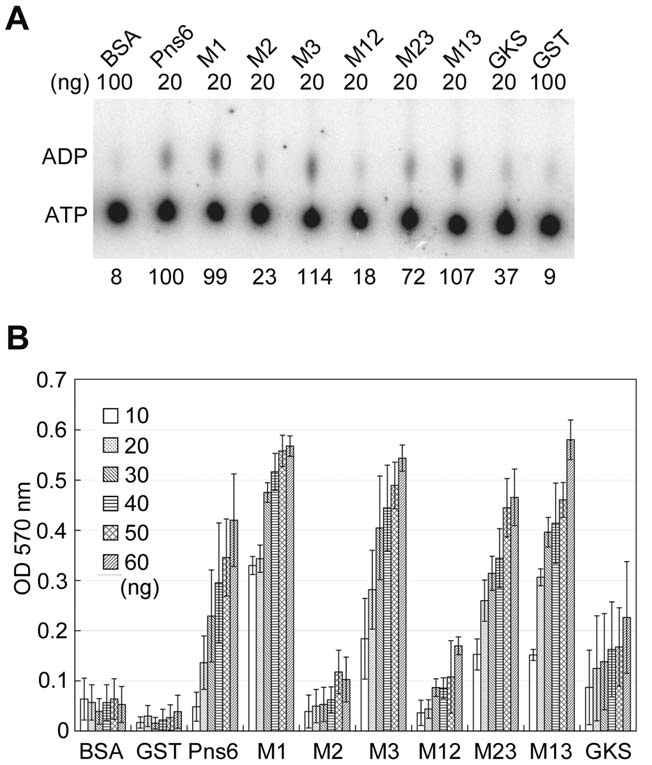
Conserved motifs and the active site of Pns6 ATPase. (A) Thin layer chromatography shows that M1 and the mutants containing M3 (M3, M23 and M13) have the strongest ATPase activity. The GKS mutant shows weaker ATPase activity than Pns6, while M2 and M12 show very little activity. BSA and GST were used as negative controls. Relative values of released ADP are presented below each lane. (B) Colorimetric malachite green assay showing the ATPase activities of the mutant proteins. All proteins were used at a range of 10 ng to 60 ng. Error bars represent standard deviations of three biological replicates.

We performed TLC and colorimetric malachite green assays with purified mutant Pns6 proteins to locate the potential ATPase activity site. Mutants M1, M3, M13 and M23 showed ATPase activity equal to or higher than that of wild-type Pns6 in TLC assays (Figure 7A). Mutants M2, M12 and GKS/AAA showed a significant reduction in ATPase activity. Consistent results with obtained from the colorimetric malachite green assays (Figure 7B). These results suggest that the ATPase activity of Pns6 resides in the C-terminal region and that GKS is a conserved ATPase motif.

## Discussion

Although our knowledge of the detailed RDV replication cycle is still very limited, it has been proposed that the this virus may have a similar lifecycle to animal reoviruses [14]. Early after infection, reoviruses become partially uncoated to form subviral particles. During this process, the viral mRNA, identical to the sense strand of the genomic dsRNA, is released from the subviral particles while the genomic dsRNA remains inside the particles. Once the viral genomic ssRNAs have accumulated to a high level and are packaged into virions, dsRNAs can be synthesized by viral RdRP (RNA-dependent RNA polymerase) inside the core. The virions can be observed in the cytoplasm at the sites where viral replication or translation have occurred [13], [14].

Virions or viral ribonucleoprotein complexes (vRNPs) can be transported through plasmodesmata into adjacent cells. In the case of RDV, it is unclear whether the virus is transported in the form of virions or vRNPs. RDV virions spread among cells of insect vectors through tubular structures composed of RDV Pns10 [22]. These tubular structures are not found in the host plant cells. In our previous report, Pns6 but not RDV virions was localized to plasmodesmata in cell walls [18]. It is possible that the diameter of the RDV virion (about 70 nm) prevents it from moving through plasmodesmata. Our previous and current data support the hypothesis that RDV may move between cells in the form of vRNPs.

Pns6 binds dsRNA in a non-sequence-specific manner. Many known MPs, such as the 30 K protein of TMV, bind ssRNA cooperatively and sequence-non-specifically [30]. Interestingly, Pns6 shows sequence preference when it binds ssRNA. Specifically, it binds preferentially to the conserved 5′- and 3′- terminal consensus sequences of the RDV genome and exhibits a stronger binding affinity for the RDV 5′- end sense strand sequence than with the corresponding antisense strand. This is a novel property unreported for other known plant viral MPs. However, a specific property of reoviruses is that their genomic RNAs contain terminal consensus sequences. In rotavirus, the 3′- terminal consensus sequence is recognized by NSP3 and is related to the translation of viral mRNA [31] while the 5′ and 3′ sequences are also recognized by viral RdRP for efficient dsRNA synthesis [32], [33]. Thus, our data also suggest the intriguing possibility that Pns6 has additional roles in viral replication and translation, an important issue to be addressed in future studies.

The ATPase activity of Pns6 may be important for RDV movement. Numerous plant virus MPs have ATPase activities [34]. In potexviruses, the ATPase activity of PVX 25 K may be required to provide the driving force to traffic viral RNA through plasmodesmata or to suppress silencing [9], [35]. The conserved GKS sequence is required for the NTP binding function of many proteins [36]. In the viral RNA helicases, which exhibit NTPase activity and have been divided into three superfamilies, the GKS motif is shared by the conserved Walker A site of SF3 helicases and the conserved segment I of SF2 and SF3 helicases [37]. This motif is present in many proteins encoded by reoviruses (e.g., VP6 of the bluetongue virus) and possesses NTPase activity [38], [39], [40]. The importance of GKS motif for Pns6 ATPase activity is consistent with findings from other viral proteins. When the GKS motif is changed to GAA in Bamboo mosaic virus ORF1, to GAS in the Hepatitis E virus helicase motif I, or to GES in the Hepatitis C virus NS3, the ATPase activities of these proteins decrease by 70% [41], [42], [43].

In summary, our analyses of RDV Pns6 uncovered the novel property of a viral MP in differentially recognizing dsRNA and ssRNA. Such property, together with the identification of the RNA binding sites and ATPase activity site in the protein, establishes a biochemical basis to enable further studies on the mechanisms of dsRNA viral MP functions in movement and perhaps also other aspects of the viral life cycle.

## Materials and Methods

### Plasmid construction

Plasmids pGEM-S6, pGEM-S7 and pGEM-S9 were digested with *BamH I* and *Sal I*, and the resulting S6/S7/S9 segments were inserted into the prokaryotic expression vector pET28a to yield pET28a-S6, pET28a-S7, pET28a-S9. Primers for constructing the S6 mutant segments are listed in Table 2. Segments M13 and GKS were generated using overlap extension PCR [44]. All resulting PCR fragments were inserted into pBluescript II KS at the *EcoR V* site to generate pBS-mutant plasmids. After digesting the pBS-mutant plasmids with *BamH I*, the resulting mutant fragments were ligated into the prokaryotic expression vector pGEX-4T-1 at the *BamH I* site to generate pGEX-mutant plasmids. The *BamH I/Xho I* fragment released from the pGEX-mutant was inserted into pET28a at the *BamH I*/*Xho I* site to produce pET28a-mutant plasmids.

**Table 2:** Primers for constructing S6 mutants.

*Primer*	*Sequence* (5′-3′)	Segment
M1F	CTTggatcccatatgGACACAGAAACTCTTTG	M1, M12, GKS, M13
M1R	ACCgaattcCCTCAGATCCCAGAATAGATTAC	M1
M2F	ATCggatcccatatgGATGATAGTAGCCATGAAGTC	M2, M23
M2R	ACTgaattcATCGGGGAAATCGTCAGAAG	M2, M12
M3F	CTTggatcccatatgATCGTTAAGATCCTCTCATATAC	M3
M3R	GTCgaattcTTTGTACACGGTAATAGCAAGTAGC	M3, M23, GKS, M13
GKSF	GTTGAGGCTGCAGCTCACAACATACTTTCTGTTG	GKS
GKSR	GTTGTGAGCTGCAGCCTCAACAAACTTTTTGTGC	GKS
M13F	GGATCTGAGGATCGTTAAGATCCTCTCATATAC	M13
M13R	GATCTTAACGATCCTCAGATCCCAGAATAGATTAC	M13

Restriction sites (*BamH I* and *Nde I* at the 5′ ends and *EcoR I* at the 3′ ends) are denoted with lowercase letters. Underlined text shows the complementary base pairs for overlap extension PCR.

### Protein preparation

*Escherichia coli* BL21 cells (TaKaRa) containing pET28a-S6, pET28a-S7, pET28a-S9 or pET28a-S6 mutant were used to produce His-tagged Pns6, P7, P9 and mutant proteins, and BL21 cells carrying pGEX-mutant constructions were used to produce GST-fused S6 mutant proteins. The cells were cultured in Luria-Bertani medium until they reached an OD_600_ of 0.6, and then the recombinant proteins were induced by adding isopropyl β-D-thiogalactoside (0.5 mM) for 2 hours at 37°C or overnight at 18°C. The soluble recombinant proteins were purified using affinity chromatography with a Ni^2+^-chelating column or a Glutathione Sepharose 4B column. The His-tagged proteins were purified in a buffer containing 20 mM TrisCl and 500 mM NaCl, pH 7.0, and the GST fused proteins were purified in a buffer containing 40 mM Tris and 50 mM NaCl, pH 8.0. The resulting proteins were used for ATPase analysis and electrophoresis mobility shift assays (EMSA).

Proteins in inclusion bodies were isolated and recovered for northwestern blotting analysis. Cells were harvested by centrifugation at 5,000 g at 4°C for 15 min and resuspended in lysis buffer (50 mM TrisHCl pH 7.0, 100 mM NaCl and 1 mM EDTA, 1% Triton X-100) at 4°C. After sonication and centrifugation at 20,000 g at 4°C for 15 min, the resulting pellet was washed once with the same lysis buffer. The washed inclusion bodies were resuspended in 6 M guanidine hydrochloride, 100 mM TrisHCl pH 7.0, 100 mM dithiothreitol, 1 mM EDTA. The solubilized proteins were separated by SDS-PAGE. The gel was stained with cold 0.5 M KCl. The respective band was then cut out, crushed, and mixed with the SDS-PAGE loading buffer. After 10 min incubation at 80°C, the sample was centrifuged for 15 min at 13,000 g and the supernatant was collected for northwestern blotting.

### Probe preparation

The sequences of the synthesized RNA probes and the templates used for the in vitro transcription are presented in Table 1. Short ssRNAs and dsRNAs were synthesized and purified via HPLC by TAKARA Biotechnology (DALIAN) Co. The MAXIscript Kit (Ambion) was used to transcribe long RNAs. The ssA-Dig probe was digoxin-labeled with the DIG RNA Labeling Kit (SP6/T7) (Roche). The radioactive probes were end-labeled with γ-^32^P-ATP using T4 polynucleotide kinase (NEB).

### Northwestern blotting and electrophoresis mobility shift assay (EMSA)

Northwestern blotting was used to detect the nucleic acid binding activity of Pns6 and its mutants as described previously [45]. The recovered proteins were separated by SDS-PAGE and then transferred to a nitrocellulose membrane. The membrane was soaked in buffer A (20 mM Tris⋅HCl, pH 7.5, 50 mM NaCl, 0.1% Triton X-100, 1 mM DTT, 1 mM EDTA, pH 8.0 and 0.02% Ficoll 400) overnight at 4°C to renature the proteins. The digoxin-labeled RNA probes or the radioactive RNA probes were then added to the buffer. After 30 min of incubation, the membranes were washed three times in buffer A for 30 min at 4°C followed by UV crosslinking. The RNA probes were detected using an anti-digoxin antibody (AP conjugated, Roche) or via autoradiography with a PE Cyclone phosphor screen scanner system.

For EMSA, RNA probes were incubated with various concentrations of proteins for 20 min on ice in a 10 µl reaction system containing 50 mM NaCl, 50 mM Tris⋅HCl, pH 7.5, and 10% glycerol. Samples were separated by native PAGE in a 9% gel in 0.5×TBE buffer for 80 minutes at 180 V, and then the gel was dried for autoradiography. In the competition assays, unlabeled RNAs were added to compete with the labeled RNA probes. The competition ability of the RNAs is related to the signal from the free RNA probes, which was quantified with ImageJ (version 1.4) software.

### Thin layer chromatography (TLC) and colorimetric malachite green assay

In the TLC ATPase analysis, we used a 10 µl reaction system containing 50 mM Tris, 5 mM MgCl_2_, 1 mM DTT, 0.5 mM ATP and the respective proteins. α-^32^P-ATP was added to the reactions at 0.05 µCi/µl. After a 30 min incubation at 37°C, 0.5 µl of the reaction product was dotted onto a PEI thin layer chromatography plate (Merck), and the plate was developed in 0.15 M LiCl and 0.15 M formic acid as described previously [6]. Images were obtained using a PE Cyclone phosphor screen scanner system.

The colorimetric malachite green assay was performed as described previously [46]. We used a 100 µl reaction system containing 50 mM Tris, 5 mM MgCl_2_, 1 mM DTT, 1 mM ATP and the respective protein. A 96-well microplate was used to analyze multiple samples. After 60 min of incubation at 37°C, 45 µl of the colorimetric mixture (0.07% malachite green, 3.7% ammonium sulfate, 2.27% ammonium molybdate tetrahydrate and 0.134% Tween-20, prepared 2 hours prior to the assay) was added to the reactions. To develop the reaction, 45 µl of 15% sodium citrate was added. The OD_570_ of the samples was read by a TECAN SUNRISE basic scanner.
